# Supplementary material for: GLUT1 overexpression enhances glucose metabolism and promotes neonatal heart regeneration
Source: Sci Rep. 2021 Apr 21;11:8669. doi: 10.1038/s41598-021-88159-x (PMC8060418; doi:10.1038/s41598-021-88159-x)
Supplement: Supplementary file 1 — Supplementary Information. [file 41598_2021_88159_MOESM1_ESM.pdf]

GLUT1 overexpression enhances glucose metabolism and promotes neonatal heart regeneration

Viviana M Fajardo[1], Iris Feng[2], Bao Ying Chen[3,5,13], Cesar A Perez-Ramirez[4], Baochen Shi[2], Peter Clark[3,5], Rong Tian[6], Ching-Ling Lien[7,8,9], Matteo Pellegrini[2,11,12], Heather Christofk[4,11,12], Haruko Nakano[2], Atsushi Nakano[2,10,11,12*]

Supplementary Figure Legends

Supplementary Figure 1. Increased glucose uptake in Glut1 transgenic heart

a. Glut1 immunofluorescent staining of *aMHC-hGLUT1* transgenic heart at P2 pre- and post-injury and P7 post injury. Note the strong sarcolemmal expression of Glut1 in Glut1 transgenic hearts (arrowheads). Scale bar= 10µm
b. Gene expression quantification by qPCR. **Left** bar graph represents Slc2a1 gene expression of hearts at P4. n = 4-5 per group *$p < 0.05$, **$p < 0.01$ **Right** bar graph represents Slc2a1 gene expression of hearts at P7. n = 4-6 per group *$p < 0.05$, **$p < 0.01$.
c. Glucose uptake of Glut1 transgenic hearts measured by autoradiography of accumulated ^{18}F-FDG activity at P2, 4, and 7.
d. Glucose uptake of Glut1 transgenic heart measured by ^{18}F-FDG activity in the autoradiography images at P2, 4, and 7. n = 1-3 for each group ** $p < 0.01$, *** $p < 0.001$
e. Blood glucose levels at P1, pre-injury, n = 20 vs 29. *$p < 0.05$ by t-test. P3, pre-injury, n = 5 for each group. P7, pre-injury, n = 9 for each group.
f. Heart weight body weight (HW/BW) ratio of wild type and Glut1 transgenic (Glut1 tg) sham control hearts. n = 5 for each group. p = n.s.
g. PCNA staining of the sections from wild type and Glut1 transgenic hearts 2 and 4 days post-injury. Sections were stained with a cardiac marker (Tnnt2; Red), proliferation marker (PCNA; Green) and a nuclear marker (DAPI; Blue). Note that PCNA staining is more abundant in Glut1 transgenic heart. Scale bars= 100 µm and 50 µm respectively.
h. **Left** graphs represent the number of cardiomyocytes from whole hearts post-injury (top) and sham controls (bottom) at P7 stage. n = 5 per group. **Right** graphs represent the number +Edu cardiomyocytes post-injury (top: n = 5 per group) and sham controls (bottom: n = 3 wild type, n = 4 Glut1 tg). *$p < 0.05$.

Supplemental Figure 2. Cardiac function of Wild type and Glut1 transgenic hearts after surgery

a. In vivo trans-thoracic echocardiography and representative M mode echocardiogram.
b. Cardiac ejection fraction at P21 and P40 stages after surgery. P21 stage, n = 3, each. P40 stage, n = 3 wild type, n = 1 Glut1 tg
c. Fractional shortening at P21 and P40 stages after surgery. P21 stage, n = 3, each. P40 stage, n = 3 wild type, n = 1 Glut1 tg

All data represent mean +/- SEM.

Supplementary Figure 3. Increased neovascularization in the border zone of Glut1 hearts after cardiac injury

Representative images of Wild type sham (a), Wild type injured (b), and Glut1 transgenic injured (c) hearts at p7. Left, (a) representative image of H&E staining, (b,c) representative image of Masson's Trichome staining. Right, immunostaining with CD31 (endothelial marker; red). (d) Quantification of neovascularization at P7 stage after cardiac injury. n = 2 wild type, n = 4 Glut1 tg

Supplementary Figure 4. RNA-seq analysis of Tnnt2^{high} and Tnnt2^{low} cardiomyocytes from Wild type and Glut1 transgenic hearts at P1

a. 2-way comparison of Tnnt2^{high} and Tnnt2^{low} cardiomyocytes from Wild type and Glut1 transgenic hearts. Note the significant increase in the number of differentially expressed genes (DEGs) between Glut1 tg Tnnt2^{high} and Glut1 tg Tnnt2^{low} cardiomyocytes.
b. Gene Ontology of DEGs among 4 populations: [1]Glut1 tg Tnnt2^{low} vs Glut1 tg Tnnt2^{high}; [2]Wild type Tnnt2^{low} vs Wild type Tnnt2^{high}; [3]Glut1 tg Tnnt2^{low} vs Wild type Tnnt2^{low}; [4]Glut1 tg Tnnt2^{high} vs Wild type Tnnt2^{high}.
c. Histogram of the forward scatter of the 4 populations. Note that Tnnt2^{low} cardiomyocytes are smaller in size in both Wild type and Glut1 transgenic hearts.

Supplemental Figure 5. Neonatal cardiac metabolite differences prior and post-surgery

a. Heatmap of metabolites before and after surgery at P2 stage comparing wild type control hearts vs Glut1 transgenic hearts and P7 stage after surgery. P2 stage groups were compared pre-injury and post-injury separately.
b. Volcano plots of metabolites at P2 stage pre-injury, n = 5 per group; P2 stage post-injury, n = 3 per group, comparing wild type control hearts vs Glut1 transgenic hearts and P7 stage post-injury, n = 3 wild type, n = 6 Glut1 tg hearts

Supplementary Table 1. Top 50 Differentially expressed upregulated genes >1.5 fold change.

Supplementary Figure 1

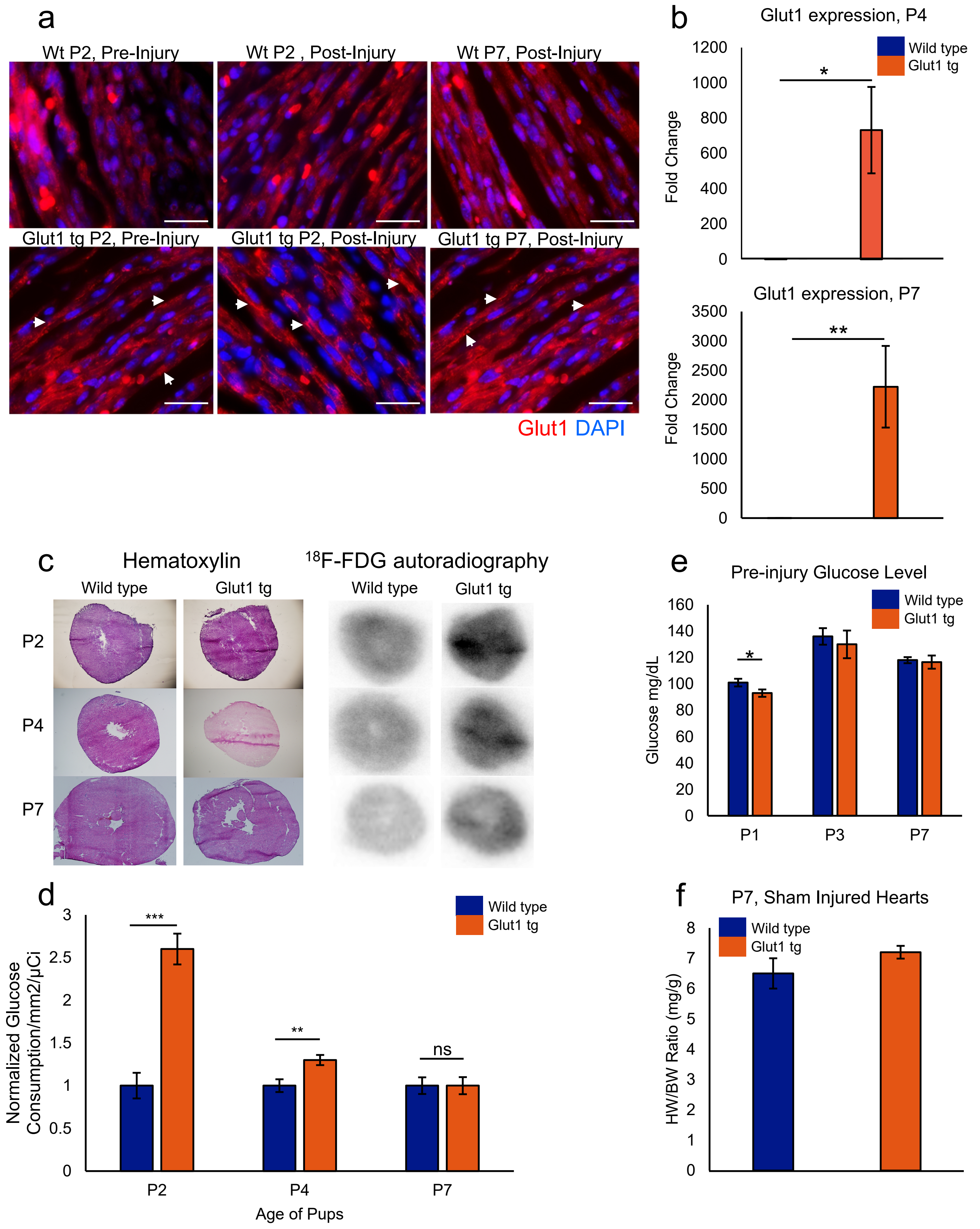

g

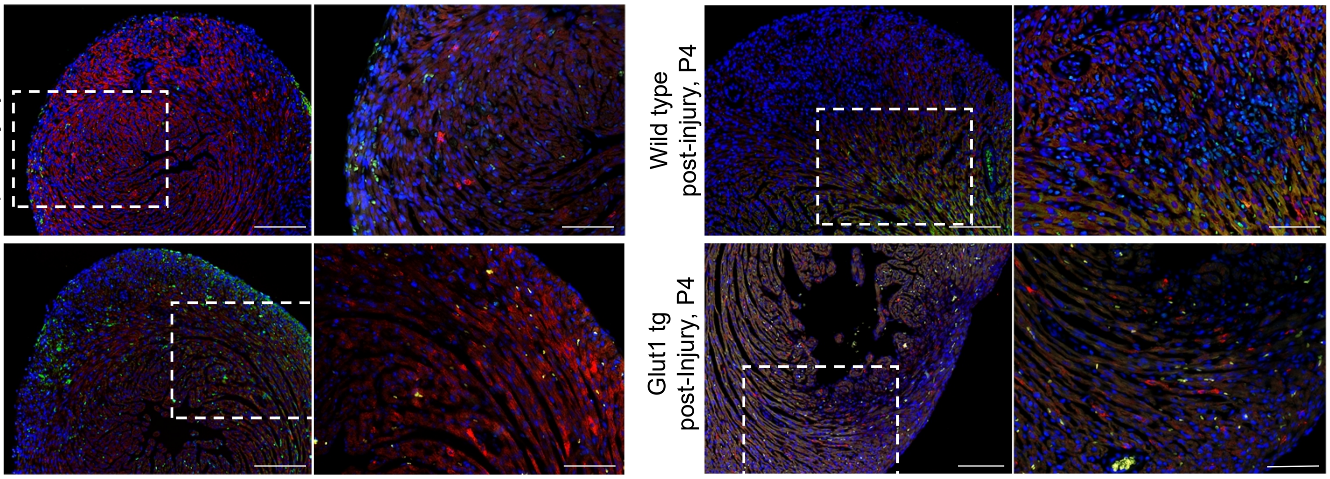
PCNA Tnnt2 DAPI
Wild type post-injury, P2
Glut1 tg post-Injury, P2
Wild type post-injury, P4
Glut1 tg post-Injury, P4

h

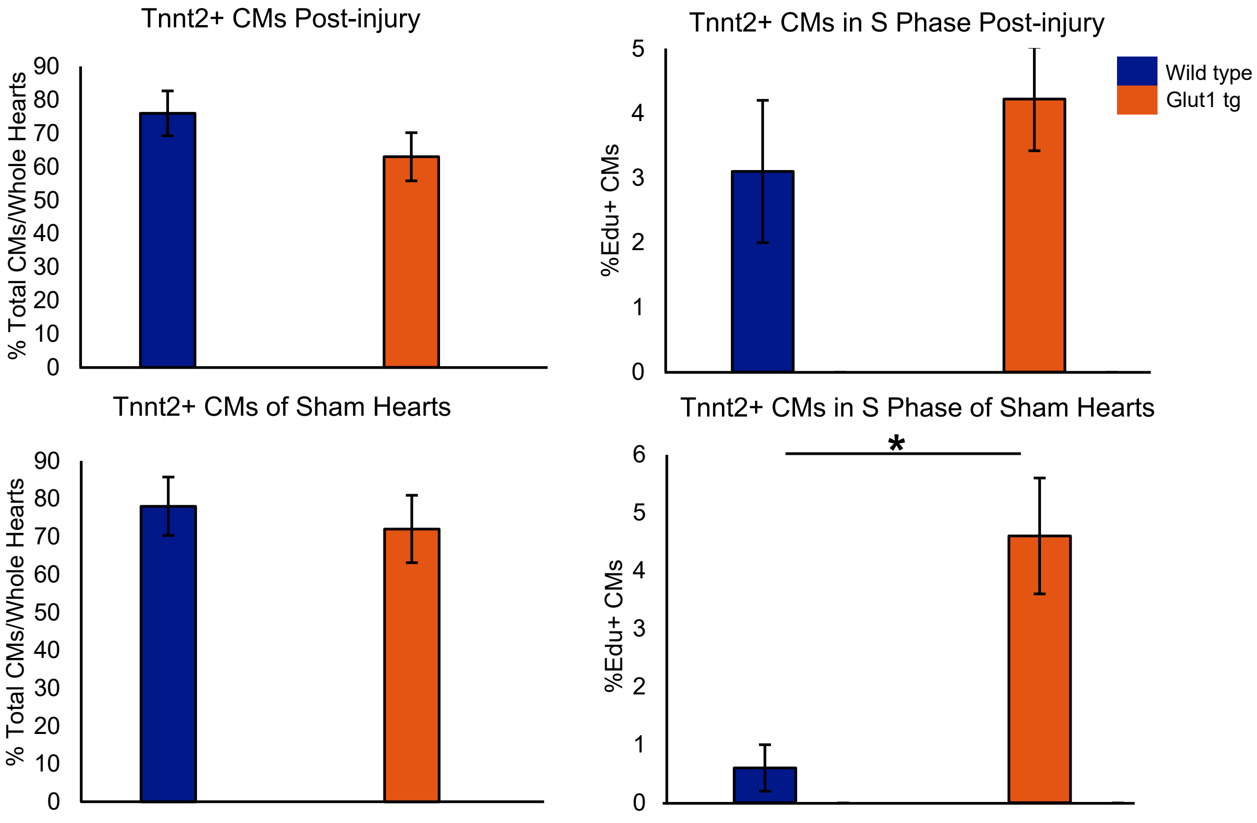
Tnnt2+ CMs Post-injury
% Total CMs/Whole Hearts
0
10
20
30
40
50
60
70
80
90
Tnnt2+ CMs in S Phase Post-injury
%Edu+ CMs
0
1
2
3
4
5
Wild type
Glut1 tg
Tnnt2+ CMs of Sham Hearts
% Total CMs/Whole Hearts
Tnnt2+ CMs in S Phase of Sham Hearts
%Edu+ CMs
6
*

Supplementary Figure 2

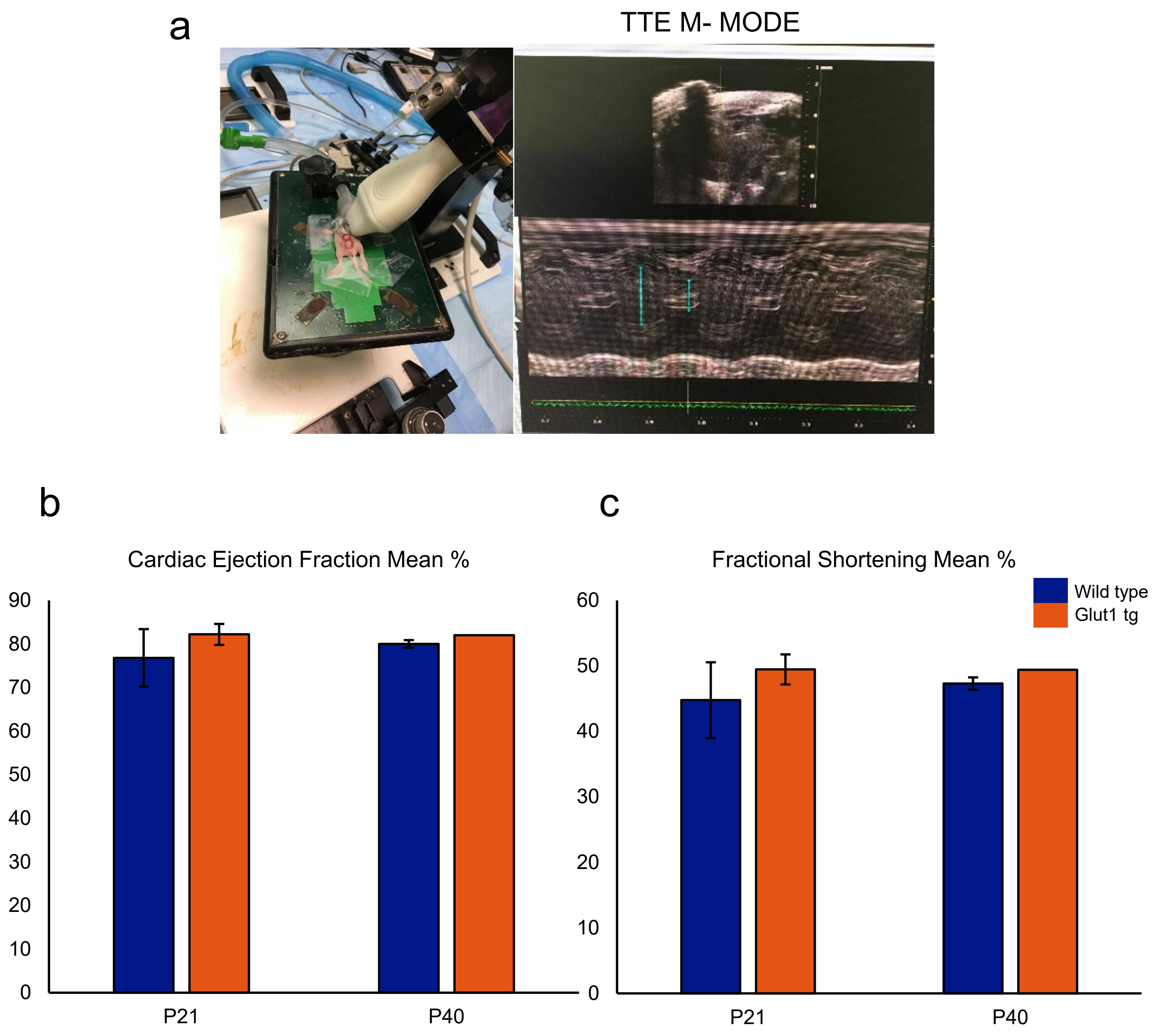

Supplementary Figure 3

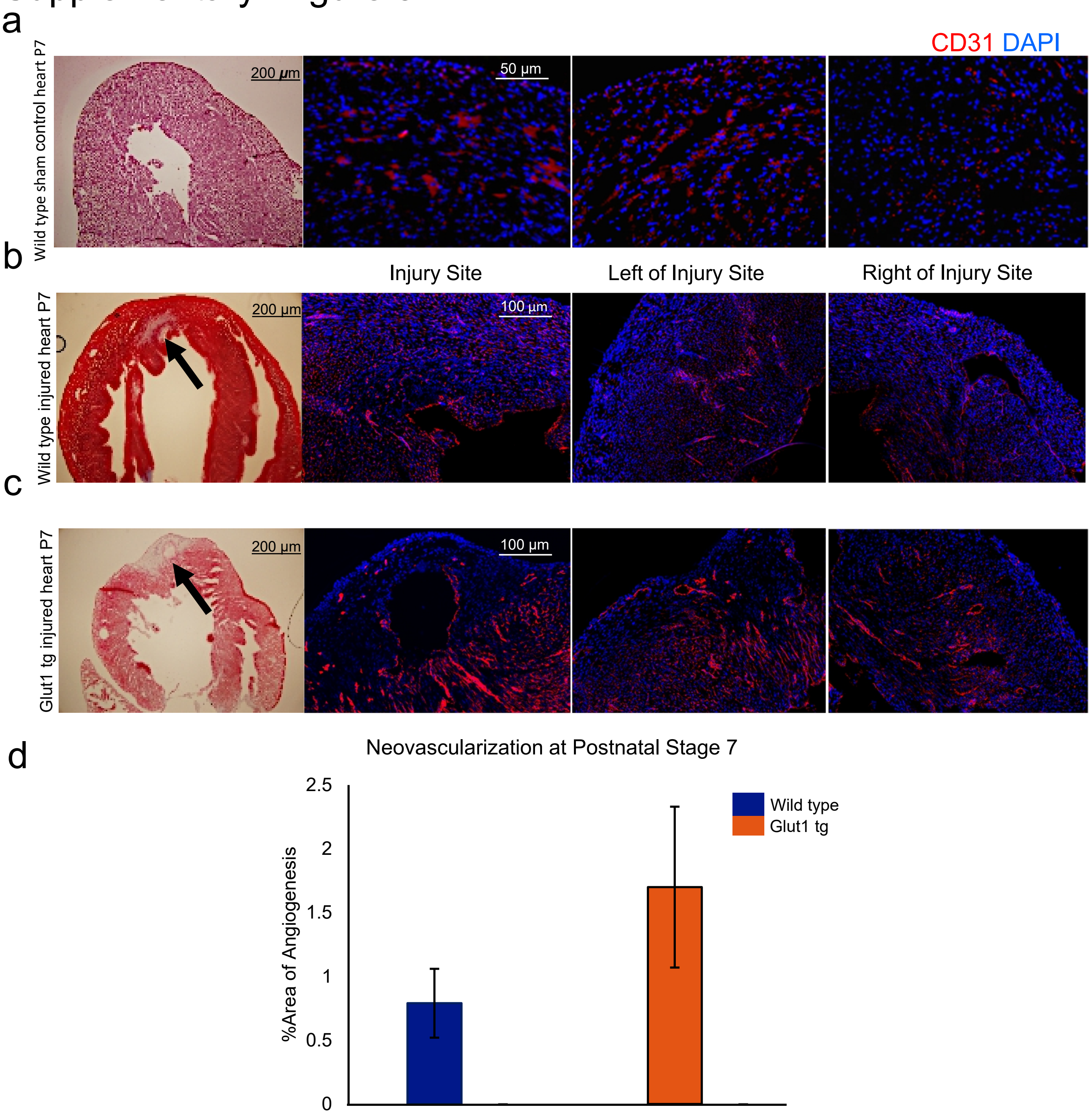

Supplementary Figure 4

a

Differentially Expressed Genes >1.5 fold change	Glut1 Tnnt2^{low} vs Glut1 Tnnt2^{high}	Wild type Tnnt2^{low} vs Wild type Tnnt2^{high}	Glut1 Tnnt2^{low} vs Wild type Tnnt2^{low}	Glut1 Tnnt2^{high} vs Wild type Tnnt2^{high}
Upregulated	918	76	68	70
Downregulated	349	107	42	82

b

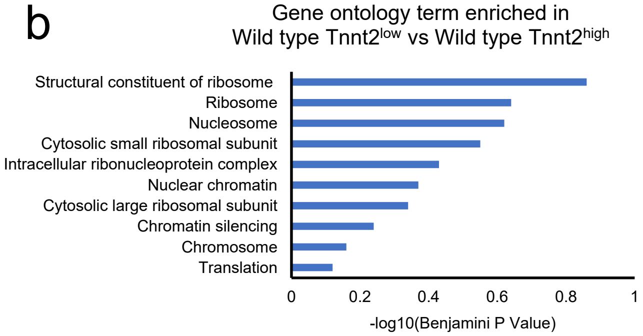

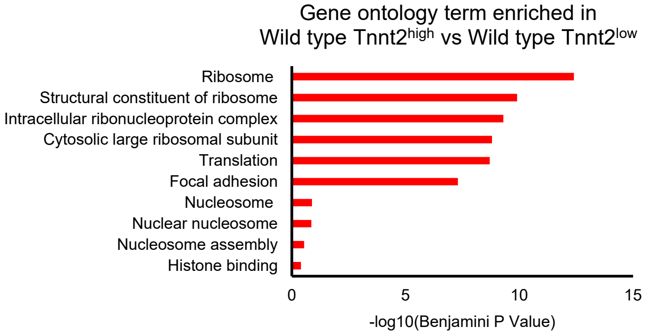

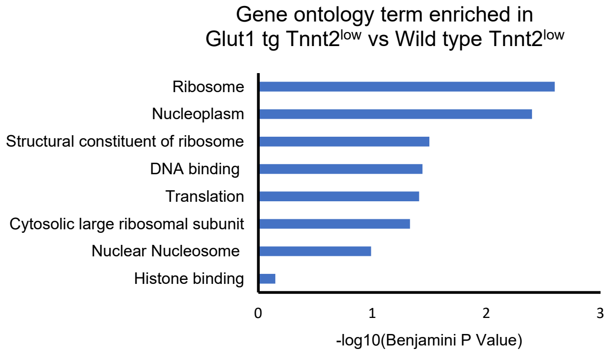

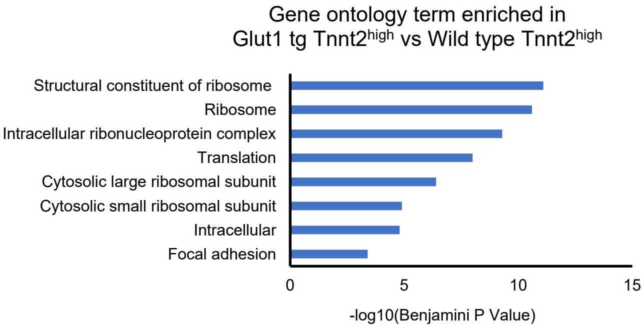

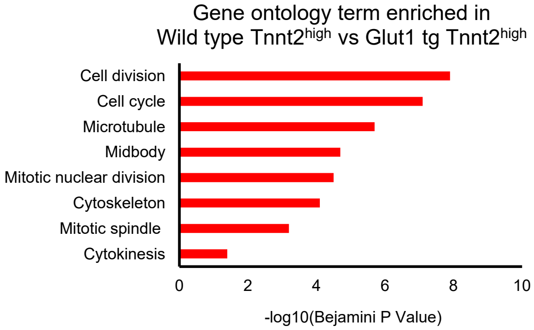

c

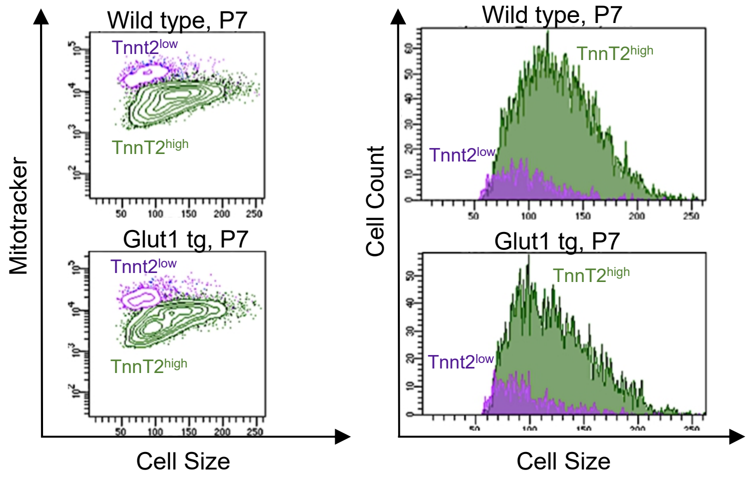

Supplementary Figure 5

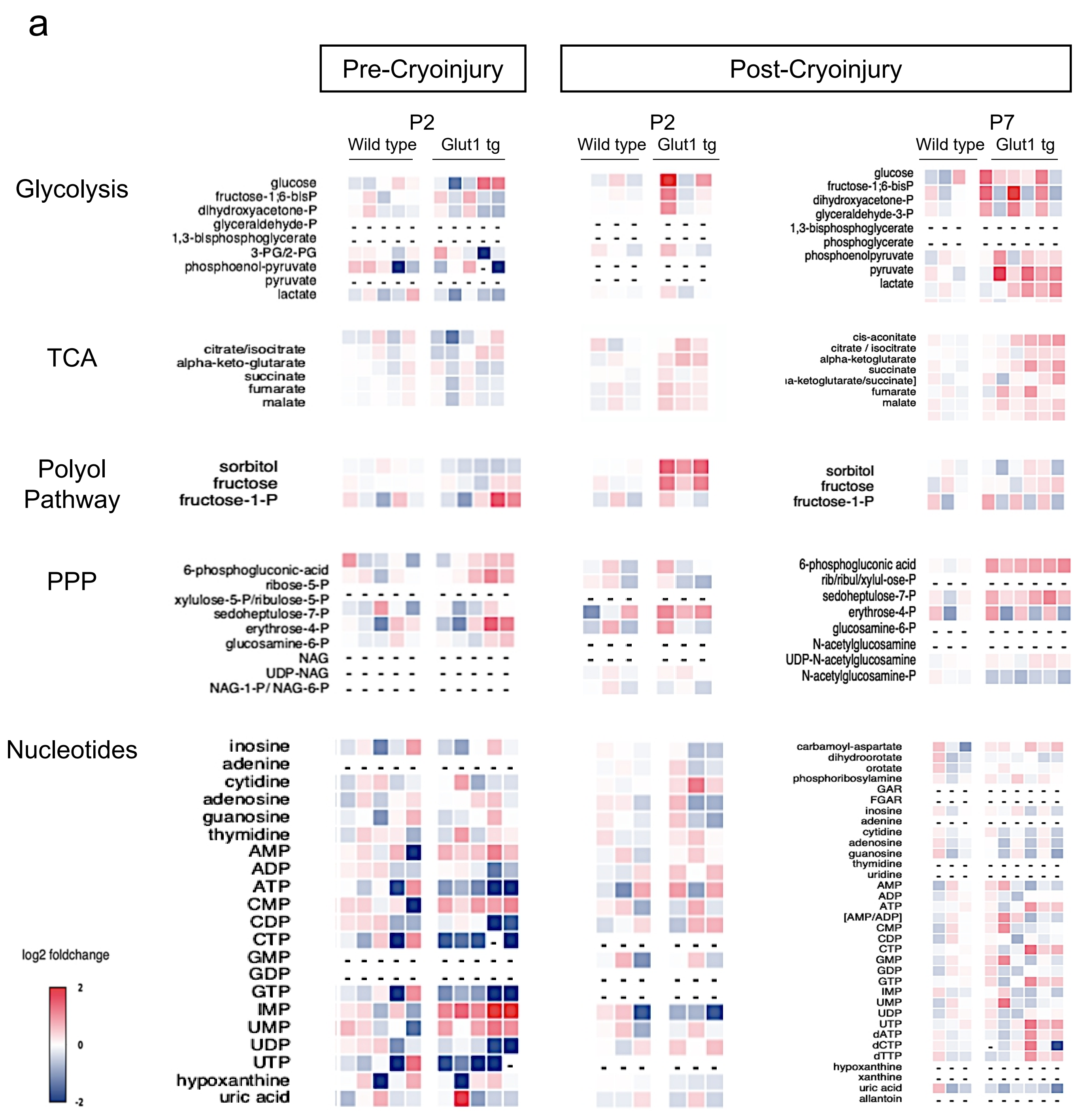

b

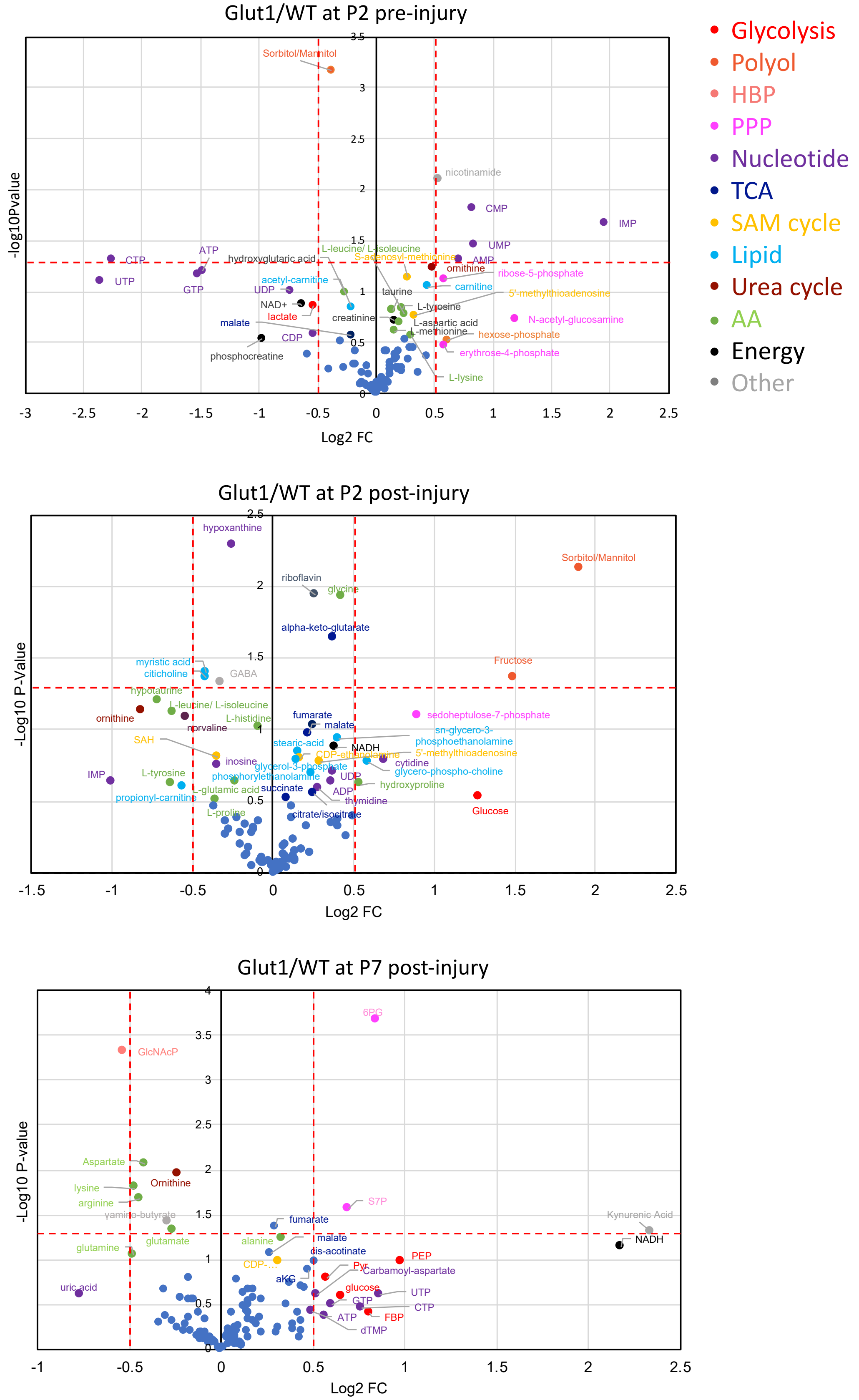
Glut1/WT at P2 pre-injury
-log10Pvalue
Log2 FC
Sorbitol/Mannitol
nicotinamide
CMP
IMP
UMP
AMP
ATP
CTP
UTP
GTP
hydroxyglutaric acid
L-leucine/ L-isoleucine
S-adenosyl-methionine
ornithine
ribose-5-phosphate
acetyl-carnitine
UDP
taurine
carnitine
5'-methylthioadenosine
NAD+
lactate
L-tyrosine
N-acetyl-glucosamine
creatinine
malate
L-aspartic acid
L-methionine
hexose-phosphate
CDP
phosphocreatine
erythrose-4-phosphate
L-lysine
Glut1/WT at P2 post-injury
-Log10 P-Value
Log2 FC
hypoxanthine
Sorbitol/Mannitol
riboflavin
glycine
alpha-keto-glutarate
myristic acid
citicholine
GABA
Fructose
hypotaurine
L-leucine/ L-isoleucine
L-histidine
ornithine
norvaline
fumarate
malate
sedoheptulose-7-phosphate
sn-glycero-3-
phosphoethanolamine
stearic-acid
NADH
SAH
5'-methylthioadenosine
inosine
cytidine
glycerol-3-phosphate
IMP
L-tyrosine
phosphorylethanolamine
UDP
glycero-phospho-choline
hydroxyproline
L-glutamic acid
succinate
ADP
propionyl-carnitine
thymidine
Glucose
L-proline
citrate/isocitrate
Glut1/WT at P7 post-injury
-Log10 P-value
Log2 FC
6PG
GlcNAcP
Aspartate
Ornithine
lysine
arginine
S7P
γamino-butyrate
fumarate
Kynurenic Acid
glutamate
alanine
malate
NADH
glutamine
dis-acotinate
PEP
CDP-...
Pyr
Carbamoyl-aspartate
aKG
uric acid
glucose
UTP
GTP
CTP
ATP
FBP
dTMP
Glycolysis
Polyol
HBP
PPP
Nucleotide
TCA
SAM cycle
Lipid
Urea cycle
AA
Energy
Other

Supplementary Table 1

Glut1 Tnnt2^{low} vs Glut1 Tnnt2^{high}	Wild type Tnnt2^{low} vs Wild type Tnnt2^{high}	Glut1 Tnnt2^{low} vs Wild type Tnnt2^{low}	Glut1 Tnnt2^{high} vs Wild type Tnnt2^{high}
Ndufab1	Ndufab1	Rps17	Hnrnpf
Mki67	Hist1h2bj	Rpl28	Ppia
Cenpf	Rpl36a	Hnrnpf	Hist1h2ac
Fosb	Rpl28	Ppia	Ddx3y
Neurl1b	Rps3a1	Cox7c	Eif2s3y
Hist1h2ac	Rps6	Eif2s3y	Cdk2ap1
Arhgap11a	Armcx3	Hist1h2bp	Eef1a1
Foxm1	Hist1h2ah	Ddx3y	Sumo2
Iqgap3	Rps8	Armcx3	Hist1h4c
Stmn1	H2afz	Btf3	Hist1h3i
Aspm	Tmem38b	Hist1h3i	Rpl23a
Ube2v1	Lsm7	Rps3a1	Tmed2
Prc1	Rpl36al	Rpl10	Cox7c
Hmmr	Kpna2	Eef1a1	Rps4x
Cenpe	Hist1h2bl	Slc2a3	Rpl29
Anln	Fosb	Rps29	Slc2a1
Top2a	Cdv3	Rpsa	Hist1h2bc
Ube2s	Tubb2a	H2afz	Rpl18
Zfp704	Erdr1	Gm6654	Slc2a3
Kif11	Tax1bp3	Uty	Uty
Ptma	Trim7	Rps10	Nsa2
Ckap2l	Ubb	Rpl36al	Hspa1b
Racgap1	Hmgb2	Rps4x	Phb
Ccna2	Eppk1	Rprl2	Tom1
Hipk2	Fos	Lsm7	H2afx
Ago2	Frmd6	Slc2a1	Wdfy1
Tnrc6c	Myh7b	Fbn1	Kdm5d
Tnrc18	Gm12504	Slc25a5	Hadhb
Kcnb1	Rab11b	Rpl37a	Acp1
Fat1	Xist	Chchd2	Arrdc4
Kmt2d	Oaz2	Rbmxl1	Rpl38
Prrc2b	Camsap1	Hist1h3e	Fras1
Map1a	Kcna4	Eln	Hist1h2bp
Bahcc1	Rbmxl1	Hspa1b	Pcna
Spen	Neat1	Rprl3	Hells
Extl3	Hist1h2ai	Erdr1	Set
Hist1h2bc	Cyb5r4	Myl12a	Fosb
Irs2	Ube2v1	Tceb2	Gm12657
Nav2	Msl2	Tubb2b	Ndufb4
Kifc1	Ccnb1	Fn1	Mid1
Cyr61	Cdon	Vwf	Myef2
Ccnb2	Gm13157	Rps15a-ps4	Pask
Ect2	Ldoc1l	Arrdc4	Socs3
Nek2	Gigyf1	Acp1	Hccs
Knstrn	Gon4l	Gm12504	Rprl2
Fos	Sirpa	Rpl35a	Cyr61
Rreb1	Hmcn1	Hist1h2ah	Napepld
Mical3	Igfbp5	Rpl36	Cyfip2
Plxna4	Cbx6	Gng5	Rps13
